# Number of Dentists in Germany

**Published:** 1890-02

**Authors:** 


NUMBER OF DENTISTS IN GERMANY.—According to Dr. Paul Borner's "Reichs-Medicinal Kalendar fur Deutschland" for the year 1888, there are 16,864 medical men and 514 dentists in practice among a population of 46,840,587 inhabitants in the German Empire, while the number of chemists' shops is 4,671, and of hospitals 2,737. In face of these figures, it may be truly aid that the dental profession is not overcrowded in the Fatherland.

A MERRY heart doeth good like a medicine, and merriment at meals is better than pepsin on the digestion.—*Sanitary Volunteer.*

